# Stemless shoulder arthroplasty: what are the benefits?

**DOI:** 10.1016/j.xrrt.2025.07.010

**Published:** 2025-08-11

**Authors:** Maximilian Russo, Christian Schoch, Rolf Michael Krifter, Claudio Rosso

**Affiliations:** a Department of Orthopaedics and Traumatology, University Hospital Basel, Basel, Switzerland; b Medical Faculty, University of Basel, Basel, Switzerland; c St. Vinzenz Klinik Pfronten, Department Shoulder and Elbow Surgery, Pfronten, Germany; d ORTHOMEDICUM, Zentrum für Gelenkserkrankungen Graz, Graz, Austria; e ARTHRO Medics, Basel, Schweiz, Universität Basel, Basel, Switzerland

**Keywords:** Stemless shoulder arthroplasty, Cuff arthropathy, Reverse shoulder arthroplasty, rTSA, Bone preservation, Metaphyseal fixation, Revision shoulder arthroplasty

## Abstract

The emergence of stemless shoulder arthroplasty marks a significant advancement, dignified to surpass traditional stemmed approaches. Stemless reverse total shoulder arthroplasty (slrTSA) shares benefits with its anatomic counterpart, including reduced complications, bone preservation, and simplified revision. In addition, it offers potential long-term osteological advantages, minimizing stress shielding and, in case of periprosthetic fractures, easier revision. Early data suggest comparable outcomes between slrTSA and stemmed implants, with future benefits. Understanding its advantages, design variations, and clinical outcomes is paramount for its seamless integration into shoulder surgery. A narrative review focused on stemless shoulder arthroplasty was conducted, retrieving recent articles spanning the last decade. Studies examining biomechanics, implantation techniques, and clinical outcomes were critically analyzed by long-term users of stemless implants. Stemless implants exhibit biomechanical advantages, adaptability to patient anatomy, bone preservation, simplified surgical techniques, and less invasive approaches. Notably, they facilitate easier handling of periprosthetic fractures and easier revisions. Clinical outcomes reveal significant improvements in functional scores and pain improvement postoperatively and are comparable to stemmed rTSA. Challenges include patient suitability concerning bone quality. Stemless shoulder arthroplasty represents a paradigm shift in shoulder surgery, with promising mid-term outcomes. However, careful patient selection based on bone quality is crucial. Stemless implants offer significant advantages, particularly for younger adults, warranting continued research and clinical evaluation to optimize patient outcomes.

In the past 10-20 years, anatomic stemless shoulder arthroplasty has become an advanced and safe technique. The stemless total shoulder arthroplasty (TSA) is meant to outnumber primary implantation numbers within 2025.^8^^,^^9^^,^^21^^,^^48^^,^^51^

Stemless shoulder prostheses use a humeral implant without a stem but with metaphyseal fixation. The technique is becoming increasingly popular, as the bone stock and the elasticity of the epiphysis and diaphysis of the humerus can be preserved in a simple and anatomically individualized situation, which could be advantageous for young and active patients in the future. The advantages lie in the reduction of intraoperative and postoperative complications due to the simpler and more individualized anatomical implantation of the prosthesis as well as the easier revision of the humerus in the event of any subsequent interventions, such as changing to an inverse configuration in the case of a rotator cuff defect or periprosthetic fracture.^32^^,^^39^^,^^53^

The first stemless shoulder arthroplasty was introduced to the European market as an anatomical shoulder implant by Biomet (Zimmer Biomet, Warsaw, IN, USA) under the name T.E.S.S., with development and initial use in France. Newer systems, such as the Eclipse 2005 (Arthrex Inc, Naples Fl, USA) or the SMR stemless 2015 (LIMA Corporate. Udine, Italy) emerged on the markets and differ significantly in the type of anchoring, the number of components, and the contact surface.^15^^,^^38^^,^^47^ Since then, many other companies have followed the idea and concept of metaphyseal anchoring. However, stemless reverse total shoulder arthroplasty (slrTSA) systems are currently not Food and Drug Administration approved in the United States.

In stemless arthroplasty, there are mainly two different designs of humeral fixation. On the one hand, the Eclipse (Arthrex Inc, Naples Fl, USA) prosthesis uses a central screw fixation, and the other option is a press-fit impaction of a humeral "core," which enables more anatomical implantation, as the surgeon is not restricted by a fixed neck-shaft angle or the anatomically variable humeral offset.^14^^,^^52^ Conversion from an anatomic to reverse configuration is paramount in shoulder arthroplasty, both in stemmed and stemless implants, respectively. For example, the Eclipse or the Icon (Depuy Synthes, Raynham, MA, USA) need to be explanted and revised with at least a short-stemmed reverse total shoulder arthroplasty (rTSA) if a revision is necessary due to TSA failure. The T.E.S.S, the SMR, the Mirai (Permedica S.p.A., Merate, Italy), and the Embrace (LINK, Hamburg, Germany) are convertible or usable for primary reverse implantation, respectively.^45^

A good and healthy humeral bone stock is essential for implanting a stemless replacement so that the component can have osseous integration after primary stable press-fit implantation. Therefore, younger patients who are active in sports generally have good to outstanding bone quality as an optimal prerequisite for a stemless prosthesis.^19^ There is no clear cutoff in age or gender as long as the bone quality and the integrity of the metaphysis is good.^42^

## Stemless reverse total shoulder arthroplasty

rTSA is a surgical procedure frequently used today to treat cuff tear arthropathies (CTA), resulting in better function and less pain. By changing the biomechanics of the shoulder by medializing and distalizing the center of rotation (COR), the biomechanical forces of rTSA allow movement of the arm by increasing deltoid recruitment and therefore shifting the primary force generator for shoulder elevation from the rotator cuff to the deltoid muscle.^25^ However, the utilization of reverse shoulder prostheses has seen a notable surge, encompassing diverse indications such as massive cuff tears without secondary osteoarthritis (OA), primary OA with posterior wear, proximal humeral fractures in the elderly and revision shoulder arthroplasty.^14^

Historically, the development of rTSA implants followed the principles set out by Grammont. The T.E.S.S group was the first to implement these principles by designing a humeral implant completely embedded into bone, below the level of the bone cut, transmitting stresses directly to the periprosthetic bone wall and therefore decreasing shear forces on the metaphysis.^40^ This concept defined the stemless "inlay" style rTSA, with a 150° humeral cut similar to Grammont's proposal. In contrast, onlay designs, where the humeral component sits on the metaphysis at the level of the humeral neck cut, increases the lateral offset, which can increase shear and bending moments at the metaphysis.^24^

Secondly, important determinant of function in rTSA is deltoid muscle recruitment. Inlay designs maintain a more medialized humeral position, which reduces deltoid tension and lowers the risk of acromial fractures. Onlay designs increase humeral lateralization, causing lengthening of the deltoid and increasing its tension and abduction moment arm, potentially improving stability and range of motion (ROM) but also increasing the risk of acromial and scapular spine fractures due to higher loading forces.^39^

The distinction between inlay and onlay designs is therefore important because it directly affects these biomechanical parameters. Commercially, very few companies have introduced slrTSA implants. LIMA and Permedica employ an inlay type of rTSA, while FX Solutions (Saddle River, NJ, USA) offers the Easytech as an onlay implant. As the numbers of implanted rTSA increases, a growing number of complications have been revealed through various studies. A particular issue of rTSA is "scapular notching," which is unique to this type of prosthesis. Lateralization of the glenoid baseplate can reduce notching and improve rotational function but may increase joint reaction forces and risk of baseplate loosening. Whereas baseplate medialization increases compressive forces at the glenoid-bone interface, which can enhance fixation but also increases the risk of scapular notching.^2^^,^^44^

In the following, we will be discussing the current literature of biomechanics, implantation technique, radiological, and clinical results.

## Materials and methods

Relevant articles focusing on stemless shoulder arthroplasty were extracted by using a thorough PubMed/MEDLINE, Web of Science, and Embase search with following keywords "shoulder arthroplasty" and/or "stemless" and/or "anatomic" and/or "reverse" and/or "rTSA" and/or "TSA," and hereby focusing on articles of the last 10 years (January 2015-January 2025). Articles were initially screened by title and abstract for relevance, with approximately 30 selected studies subjected to full-text review, of which most of the articles discussing stemless total shoulder arthroplasty and slrTSA, focusing on clinical outcomes, indications, and complications. While a few studies with a primary focus on biomechanical outcomes were included. To synthesize current knowledge and provide a comprehensive overview of stemless shoulder arthroplasty, a narrative review approach was adopted.

### Results and discussion

The following paragraphs discuss the advantages of stemless implants, be it anatomic or reverse. The main focus is on clinical results of anatomical implants because there is still a lack of data for stemless reverse implants. In addition, the development of stemless reverse implants is discussed.

#### Better biomechanics (less stress shielding)

In a finite element study, Razfar et al showed that shortening the stem length resulted in humeral stresses that closely resembled the intact stress distribution in the proximal cortical bone.^43^ Conversely, contrasting trends were observed in the proximal trabecular bone, likely due to variations in load transfer associated with using shorter stems. Consequently, the findings propose that stemless implants can reduce stress shielding. In the study by Baumgarten et al and Magone et al, radiographic evidence of stress shielding was found in the short-term follow-up for stemmed, but not for stemless arthroplasties.^7^^,^^38^

Despite these findings in finite element models, the clinical reality shows that up to 40% of the patients exert signs of stress shielding without signs of loosening.^2^ This discrepancy likely arises from differences in modeling assumptions vs. real-world biological responses.

#### Anatomic adaptability

The use of a stemless humeral component allows greater adaptability to variations in patient anatomy through metaphyseal fixation, avoiding the constraints of a fixed neck-shaft angle or diaphyseal canal mismatch. In anatomic total shoulder arthroplasty, this improved fit can potentially lead to more natural joint reconstruction. However, in rTSA, which is inherently noanatomic, scapular notching is primarily influenced by prosthetic design factors such as the COR position and neck-shaft angle, rather than the fixation method (stemmed vs. stemless). Therefore, while stemless designs may offer surgical advantages, any impact on scapular notching would need to be directly related to changes in COR lateralization, implant sizing, or humeral inclination, rather than the absence of a diaphyseal stem itself.^11^^,^^23^^,^^30^

In a radiological follow-up of 69 patients, Kadum et al showed that stemless implants can help restore the ideal humeral positioning for anatomic prostheses. The reverse prostheses do not need to fulfill this, but it seems possible to adapt to post-traumatic deformities or the individual's anatomy by changing the resection angle.^30^ Berth et al compared the ROM in patients with stemless or stemmed reverse shoulder prostheses over two years. They found that patients with stemless prostheses showed a slight, though not statistically significant, improvement in ROM compared to those with stemmed prostheses.^10^

In a finite element study, Cunningham et al showed that keeping the resection flat (around 150°-140° shaft-neck angle) for the best primary stability is crucial.^18^ So, in most cases, stemless implantation may lead to better radiological results, but sometimes this advantage might be an issue in some problematic cases. In the senior authors' own series, scapular notching was found in only 16% of cases.^44^

#### Reduced bone loss and easier revision

Stemless designs often involve a less invasive surgical technique: the absence of a humeral stem eliminates the need for preparing the humeral canal, potentially resulting in less disruption to the bone and the surrounding tissues.^4^^,^^49^ This, on the other hand, makes the implantation of glenoidal component more challenging due to less space/retractor positioning. In a case report, Schoch et al, showed that a stemless anatomic implant can be removed and revised with another stemless implant if the humeral fixation has a good bony fixation.^46^ If a stemless anatomic prosthesis must be revised because of rotator cuff failure, it is supposed that stemless arthroplasties are easier manageable to revise with a short or standard stemmed implant. If a stemmed implant is used primarily, the revision is done with an even longer implant, sometimes the need of shaft osteotomies and osteosynthesis/cerclages.^46^ Holschen et al outlined distinctions between primary implants with stems and those without regarding clinical outcomes following revision to rTSA or rTSA, respectively.^29^ In total, 44 subjects that secondarily received a stemless implant demonstrated better postoperative Constant Score (CS) results compared to those treated with a stemmed implant (67.5 vs. 50.9, *P* = .03) at mean follow-up of 24 months. The authors concluded that conversions from stemless primary implants to rTSA/rTSA result in superior shoulder function when compared to stemmed primary implants. They posited that these findings underscore the advantages of stemless primary implants, likely attributed to a less invasive revision process that concurrently preserves humeral bone stock.^17^

#### Simplified surgical technique

Overall, the surgical technique is more straightforward, with fewer steps in stemless arthroplasty. This may result in shorter operating room time.^41^ It is believed that shorter surgical time results in lower infection rate, less blood loss, and better clinical outcomes.^40^ Until now, all comparative and single-arm studies show that the clinical results are at least as good as those of a stemmed rTSA.^6^^,^^10^^,^^26^^,^^28^^,^^36^^,^^37^^,^^47^ One has to bear in mind that although there are fewer steps to perform, preserving the bone during the operation might be more challenging to get perfect access to the glenoid as working within a smaller area makes it harder to achieve a good fit for the implant. We routinely perform preoperative 3D–computed tomography (CT) planning for shoulder replacements, primarily focusing on the glenoid component, which does not differ between stemless and stemmed rTSA. However, for the humeral component, we have found that 3D–CT planning offers limited benefit. Instead, intraoperative sizing, like the approach used with stemmed prostheses, provides a more accurate assessment. In the absence of a guiding stem, accurate implant alignment relies simply on the surgeon's skill and precision. To support proper positioning, 3D virtual motion imaging can assist preoperatively, while intraoperative fluoroscopy may be used to verify alignment after implantation.^48^

#### Easier handling of periprosthetic fractures

Periprosthetic fractures usually occur as a result of low-energy trauma in older patients. By preserving the diaphysis when using a humeral component without a shaft, the bone injuries usually appear benign in the metaphysis, with conservative treatment options or simple revision using standard implants compared to complex, risky procedures with extra-long–stemmed prostheses.^32^

In a study by Levy et al, most fractures were metaphyseal and were treated conservatively with good clinical and radiological outcomes.^35^ In a study by Anderson et al, periprosthetic fractures were surgically treated by open reduction and internal fixation or prosthesis revision. They concluded: "Periprosthetic fractures around the humeral part of a TSA are a difficult problem with complex decision-making, complications are common, and reoperations are required in 19% of cases. Over 50% of all cases had loose stems requiring revisions”.^5^

Natera and Levy were able to show that when comparing revisions of intraoperative and postoperative complications of stemmed and stemless implants, there was a significantly more complicated procedure in the group of stem-guided prostheses, with longer operation times, necessary humeral osteotomy or treatment with allografts, and a significantly higher number of intraoperative fractures.^35^

In a follow-up study of humeral revisions by Cil et al, 17% of the revised shoulders had iatrogenic intraoperative humeral fractures, 23% had cement extrusions, and 11% required early further revision procedures.^16^

#### Functional scores

SlrTSA demonstrated noteworthy improvements in functional scores across all included studies (Table I)^31^^,^^39^^,^^44^^,^^50^^,^^52^ However, the diverse use of different functional scales among studies makes direct result comparisons challenging. Kelly et al provided the latest systematic review on slrTSA in 2025.^31^ They included 15 studies with a total of 657 shoulders in 648 patients who underwent slrTSA. The mean sample size per study was 44 shoulders (range = 12 to 115). The weighted mean follow-up duration was 45 months (range: 24-102 months). Five different stemless implants were reported: T.E.S.S and Easytech FX, SMR, Verso (Biomet, Bridgend, UK), and Comprehensive Nano (Zimmer Biomet, Warsaw, IN, USA). They found that slrTSA/TSA consistently exhibited substantial enhancements in ROM across all examined studies (*P* < .05). The weighted mean flexion improved from an average of 83° before surgery to 135° after the procedure. Likewise, abduction increased from a preoperative average of 78°-125° postoperatively. Notably, different measurement systems, such as point systems, degrees, and body parts, were inconsistently utilized for assessing internal and external rotation. However, all authors reported significant improvements in both internal and external rotation. The CS emerged as the most frequently employed functional score in 12 studies. Preoperatively, the weighted mean CS was 31.6, which substantially increased to 65.4 postoperatively. The American Shoulder and Elbow Surgeons (ASES) score improved from 37.1 to 78.6 across 4 studies and the Quick Disabilities of Arm, Shoulder and Hand improved from 58.3 to 24.7. Three studies have directly compared stemless and stemmed rTSA implants, focusing on postoperative outcomes, surgical outcomes, and complications.^1^^,^^30^^,^^39^ In all of these studies, no significant differences in CS,^39^ ASES,^1^^,^^39^ ROM^1^^,^^30^^,^^39^, and visual analog scale scores^30^^,^^39^ were observed between stemless and stemmed groups. In terms of surgical time, Moroder et al found that stemless implants were associated with significantly shorter operative times (80.5 minutes vs. 109.5 minutes; *P* < .001). However, the same study reported a significantly longer hospital stay for the stemless group (11.8 vs. 7.9 days; *P* = .006), a finding not explained by the authors.^39^ Overall, no significant difference in overall postoperative complication were found in this comparative studies.

**Table I:** Relevant studies.

Author	Year	slRTSA implant	N	FU (mo)	Key outcomes	Humeral related complications
Ballas and Beguin^6^	2013	T.E.S.S	56	59	Preoperative vs. postoperative: CS: 29 vs. 62 • Oxford Shoulder Score: 46 vs. 17 • Anterior active elevation: 79° vs. 140° • External rotation: 13° vs. 45°	1 humeral bone crack without consequence
Kadum et al,^30^	2014	T.E.S.S	16	39	Significant improvement in functional outcome and reduction of pain in stemless and stemmed. No significant difference between stemless and stemmed.	2 cases of early postoperative instability due to corolla displacement, needing revision
Teissier, et al,^50^	2015	T.E.S.S	87	41	Significant improvement of CS, pain, and ROM	No complications
Von Engelhard et al,^20^	2015	T.E.S.S	65	18	Significant increase in CS and DASH, no significant difference between both groups	1 case of aseptic humeral loosening (revised with stemmed rTSA)
Levy et al,^35^	2016	IDO/Verso	98	50	Significant improvement of CS, pain was rated mild or none (96.9%), high satisfaction scores	6/98 humeral related complications (6.1%), only 1 requiring revision surgery
Moroder et al,^39^	2016	T.E.S.S	24	34.2	No significant change between groups in CS, ASES, SSV, pain level, patient satisfaction, strength, and ROM; better ROM in stemless group	1 traumatic dislocation; radiolucent lines (RLL) in 12.5%—clinically irrelevant
Beck et al,^8^	2019	T.E.S.S	47	101	Significant improvement of CS, quick-DASH, VAS, and ROM	Overall complication rate: 17.2% (5 revisions) scapular notching in 72.3%
Leonidou et al,^33^	2019	Verso	36	36	Significant improvement in functional outcome and reduction of pain in stemless and stemmed.	2 traumatic periprosthetic fractures, treated conservatively
Schoch et al,^47^	2021	LIMA	56	29.3	Significant improvement of CS and active ROM; mean SSV at 84.27%	1 complete loosening of the humeral component without reoperation
Ajibade et al,^3^	2022	T.E.S.S or Verso	430	6.4-101.6	Significant improvement ASES, quick-DASH, CS, VAS, and ROM	Overall complication rate: 11.2%; 1 case of humeral component loosening (0.2%)
Galhoum et al,^22^	2022	Nano (Zimmer)	15	40.1	Significant improvement in VAS, satisfaction, and ASES	Overall humeral components-related revision: 13%
Rosso et al,^44^	2023	LIMA	26	46.8	Significant improvement in ASES score, SSV, Quick-DASH, VAS	RLLs detected in 7/25 implants (28%); asymptomatic, no loosening or migration
A'Court et al,^1^	2024	LIMA	60	24	Comparable clinical and radiographic outcome between stemless and stemmed RSA	Implant subsidence in 2 stemless RSA patients (6.7%) (treated conservatively)
Choi CH et al,^12^	2024	LIMA	28	24	Significant improvement in elevation and internal rotation, as well as ASES and KSS scores	No humeral loosening or fracture.

*SlrTSA*, stemless reverse total shoulder arthroplasty; *CS*, Constant Score; *SSV*, Subjective shoulder value; *ROM*, range of motion; *VAS*, visual analog scale; *KSS*, Korean Shoulder Scoring; *RLLs*, radiolucent lines; *ASES*, American Shoulder and Elbow Surgeons; *DASH* (score), Disabilities of Arm, Shoulder and Hand; *FU*, follow-up; *rTSA*, reverse total shoulder arthroplasty.

The primary indications for surgery were CTAs (≈71%), OA (≈12%), rheumatoid arthritis (≈6%), proximal humerus fracture sequelae (≈6%), and others (≈5%). The included studies addressed postoperative pain, consistently reporting significant improvements. The overall complication rate for slrTSA was 13.4%. Across all studies, implant loosening was reported in 3.1% of cases, while dislocation occurred in 1.2%. In the senior author’s own series on slrTSA with a 46 months of follow-up a significant improvement was noted in the ASES score (55.9-85.6, *P* < .001), subjective shoulder value (44.3-85.3, *P* < .001), Quick Disabilities of Arm, Shoulder and Hand score (40.6-17.8, *P* < .001), visual analog scale pain score (4.6-0.9, *P* < .001), and ROM in flexion (66-154, *P* < .001), as well as in the absolute (44.1-83.1, *P* < .001) and relative CS (62.1-111.9, *P* < .001).^44^ Complications were reported in 9 patients. One required revision surgery (3.8%) because of shoulder instability due to prior surgeries and thus poor soft tissue quality. Three patients had temporary dysesthesias in the hand, all of whom fully recovered at final follow-up. Two developed significant postoperative hematomas; one needed surgical drainage, during which a torn subscapularis tendon was repaired. No implant-related complications were observed during follow-up.^44^ In conclusion, slrTSA seems to provide the comparable clinical outcomes compared to stemmed implants with the advantages of bone stock preservation and easier revision.

### Limitations of stemless reverse implants

While slrTSA implants offer several advantages over traditional stemmed designs, including reduced stress shielding and potential biomechanical benefits, they are not without limitations. Understanding and addressing these limitations are crucial for optimizing patient outcomes and advancing the field of shoulder arthroplasty.

#### Bone quality

Stemless humeral components largely rely on a secure fixation to the metaphyseal cancellous bone, avoiding diaphyseal fixation and cortical contact. These components are recommended for patients with strong metaphyseal bone, given its lower density compared to cortical bone. In a study led by Churchill et al, they introduced a surgical technique termed the "thumb test," which entails manually pressing the cut surface of the humeral neck with the thumb to assess bone quality.^15^

An additional approach to assess patient suitability for stemless fixation involves utilizing preoperative CT scans. These scans measure bone density in both the proximal diaphysis and the cancellous and cortical metaphysis, offering an alternative to bone mineral density measured by Dual-energy X-ray Absorptiometry scans. Obtaining a value on the Hounsfield scale, a quantitative measure used to describe radiodensity, allows differentiation between different types of body tissues and materials based on their density characteristics.^6^ In these investigations, bone Hounsfield Unit (HU) measurements serve as indicators. HU values below 100 signify osteoporosis, while values ranging from 100 to 160 indicate osteopenia. Conversely, HU values exceeding 160 are indicative of normal bone mineral density.^53^ Gregory et colleagues had shown that patients requiring stems were identified with densities below 20 HU through preoperative CT scans, leading to the establishment of this threshold for distinguishing between high and low metaphyseal cancellous bone density. A density below 20 HU indicates a 17.8 times higher likelihood of requiring a stemmed component.^34^ As published by Levin et al in 2022, a cutoff value of 14.4 HU yielded a sensitivity of 95% and a specificity of 100%. In addition, a threshold of 1.41 for the Deltoid Tuberosity Index resulted in a high sensitivity of 98% and a specificity of 60%. As of now, there is no test to preoperatively determine bone quality. However, the authors are working on preoperative measurements to assess bone quality before the operation.

#### Bone stock

The benefit of a stemless implant is its ability to secure the humeral component without the necessity of preparing the humeral diaphysis. Put simply, the positioning of the humeral head can be achieved irrespective of the shape of the humeral diaphysis. However, of note, an adequate bone stock is required for this procedure. In cases of acute proximal humerus fractures where there is insufficient bone stock, stemmed prostheses are utilized.

Stemless implants, owing to their isolated epiphyseal fixation, theoretically pose a greater risk of loosening compared to stemmed implants due to progressive peripheral osteolysis from polyethylene wear particles. In addition, proximal osteolysis can exacerbate due to stress shielding in the presence of a stem. Orthopedic companies and surgeons, akin to total hip arthroplasty trends, have shifted towards using short stems. However, the efficacy of this compromise relative to stemless designs remains dubious, lacking a comparable advantage in bone preservation and still harboring potential fixation inadequacies compared to standard stems.^19^ Mini-stem designs, while facilitating easier extraction in revision cases, are not optimally suited for metaphyseal bone integration.

## Conclusion

Mid-term outcomes of stemless shoulder arthroplasty featuring epiphysometaphyseal fixation are on par with those of stemmed counterparts. Since 2005, numerous orthopedic device manufacturers have introduced stemless implants for TSA, yet only a few have ventured into stemless implants for rTSA. Further long-term data are necessary to assess the efficacy of these implants.^27^

Based on the experience and the long-term success achieved with the T.E.S.S design, and the promising mid-term results of the LIMA SMR, we advocate for the use of stemless anatomic or reverse (inlay) implants for shoulder replacement, provided that there is adequate quantity and quality of bone stock. In particular, we consider stemless rTSA indicated in cases of CTA, particularly in younger patients with high functional demands and longer life expectancy. In these cases, the bone preserving nature of stemless designs offer a relevant advantage. as Stemless designs avoid diaphyseal stem placement, which may facilitate revision and reduce the risk of stress shielding and complex periprosthetic fractures.^13^

## Disclaimers

Funding: No funding was disclosed by the authors.

Conflicts of interest: Dr Rosso is a consultant for Lima Corp. and received research support from Arthrex Inc, Smith and Nephew Inc, Zimmer Biomet and Stryker; serves in the European Shoulder Associates Board of ESSKA; and is president of the shoulder and elbow expert group of Swiss Orthopedics. Dr Krifter is consultant for Lima Corp., Arthrex and Zimmer Biomet and president of GOTS Austria. The other authors, their immediate families, and any research foundation with which they are affiliated have not received any financial payments or other benefits from any commercial entity related to the subject of this article.
